# *Lonicerae japonicae flos* Polyphenols Attenuate Inflammation-Related Ferroptosis and Gut Microbiota Dysbiosis in LPS-Induced Acute Lung Injury in Mice

**DOI:** 10.3390/nu18132048

**Published:** 2026-06-23

**Authors:** Yingjian Guo, Chuangchuang Wang, Hongjing Dong, Tao Li, Chuanzhi Kang, Xiao Wang, Jinqian Yu

**Affiliations:** 1 College of Food Science and Engineering, Qilu University of Technology (Shandong Academy of Sciences), Jinan 250353, China; 2 Shandong Engineering Research Center for Innovation and Application of General Technology for Separation of Natural Products, Shandong Analysis and Test Center, Qilu University of Technology (Shandong Academy of Sciences), Jinan 250014, China; 3 State Key Laboratory for Quality Assurance and Sustainable Use of Dao-di Herbs, Institute of Chinese Materia Medica, Beijing 100700, China

**Keywords:** *Lonicerae japonicae flos*, polyphenols, acute lung injury, gut microbiota, ferroptosis, fecal microbiota transplantation

## Abstract

**Background/Objectives**: Acute lung injury (ALI) currently lacks safe and effective therapeutic strategies with low toxicity. *Lonicerae japonicae flos*, a traditional herb and functional food, contains polyphenols as its principal active components. This study investigated whether *Lonicerae japonicae flos* polyphenols (LJP) could exert protective effects against lipopolysaccharide (LPS)-induced ALI in mice. **Methods**: Eighty-four male C57BL/6J mice were randomly divided into seven groups and treated daily for 7 days with LJP (200, 100, or 50 mg/kg), liproxstatin-1 (10 mg/kg), dexamethasone (5 mg/kg), or saline (control and model groups). Subsequently, another thirty-six mice were used for the fecal microbiota transplantation (FMT) experiment. All groups except the control group received intratracheal instillation of LPS (5 mg/kg) to induce ALI. **Results**: LJP treatment significantly ameliorated lung histopathological damage and gut microbiota dysbiosis. Lung proteomics analysis revealed the enrichment of the NF-κB and ferroptosis pathways. Mechanistically, LJP downregulated pro-inflammatory factors (IL-6, TNF-α, and IL-1β) by suppressing activation of the TLR4/MyD88/NF-κB pathway. Meanwhile, LJP upregulated SOD and GSH levels, thereby suppressing the accumulation of ROS, GSSG, Fe^2+^, and MDA, which were closely related to the activation of the Nrf2/HO-1 and Sirt3/Nrf2/GPX4 pathways. Furthermore, LJP modulated the gut microbiota and promoted short-chain fatty acid (SCFA) production by elevating the relative abundance of *Akkermansia muciniphila* and *Faecalibaculum*. Intriguingly, FMT results confirmed that the LJP-derived gut microbiota markedly alleviated lung tissue injury and intestinal barrier damage in ALI mice. **Conclusions**: This study demonstrated that LJP could reshape the gut microbiota to enhance the production of SCFAs and inhibit inflammation-related ferroptosis in ALI mice.

## 1. Introduction

Acute lung injury (ALI) is a critical condition associated with substantial morbidity and mortality, characterized by severe respiratory distress and refractory hypoxemia [1]. The pathogenesis of ALI is complex, typically triggered by bacterial or viral infections as well as trauma [2]. Current therapeutic strategies primarily rely on glucocorticoids and antibiotics, such as methylprednisolone and amoxicillin [3]. However, prolonged drug administration carries the risk of drug resistance, organ toxicity, and adverse effects [4]. Due to the limitations of these drugs, safe and effective therapeutic strategies with low toxicity are urgently needed, especially natural food-derived compounds as a preventive treatment for ALI [5].

*Lonicerae japonicae flos*, the dried flower bud of *Lonicera japonica* Thunb., has been widely used as a medicinal and edible herb [6]. It contains a variety of constituents, such as polyphenols, flavonoids, and iridoid glycosides. Among these, *Lonicerae japonicae flos* polyphenols (LJP) are considered the principal bioactive components and possess anti-inflammatory, antibacterial, and antiviral properties [7], which enable their application in ameliorating multiple diseases, such as influenza and colitis [8,9]. Moreover, polyphenols possess considerable potential in lung protection. For instance, *Nymphaea candida* polyphenols have been shown to prevent ALI by regulating the intestinal microbiota and TLR4/NF-κB signaling pathway [10]. Curcumin, as a polyphenol component, alleviates sepsis-induced ALI by inhibiting the TLR4/NF-κB signaling pathway [11]. Hence, further investigation is warranted to evaluate the anti-ALI efficacy of LJP from a new perspective.

Proteomics employs a comprehensive approach to elucidate drug-induced dynamic changes in proteins by comparing protein expression profiles in cells or tissues before and after drug treatment, enabling the identification of differentially expressed proteins and potential therapeutic targets [12]. Integrating gut microbiota analysis with proteomic profiling can thus provide novel insights into the mechanisms by which LJP exerts its protective effects. Fecal microbiota transplantation (FMT) is a technique that alters the composition of the recipient’s intestinal microbiota to attain therapeutic effects [13]. Dysregulation of the gut microbiota and increased intestinal permeability are closely linked to systemic inflammation and ALI development [14]. Gut-derived microbial metabolites and bacterial translocation can exacerbate lung injury [15], which involves multiple interrelated pathological processes, including the inflammatory response, oxidative stress, lipid peroxidation, and ferroptosis [16,17]. Emerging evidence also highlights the role of microbial metabolites in ALI mitigation [18]. Elevated short-chain fatty acid (SCFA) levels, modulated by the gut microbiota, inhibit the NF-κB signaling pathway in alveolar macrophages, thereby attenuating pulmonary inflammatory responses [19].

Therefore, we aimed to determine whether LJP could mitigate LPS-induced ALI by modulating the gut–lung axis. We hypothesized that LJP would reshape the gut microbiota to increase SCFA production, and inhibit inflammation-related ferroptosis. We established an in vivo ALI model, assessed lung histopathology and inflammatory biomarkers, performed lung proteomics and gut microbiota analysis, and conducted FMT validation. These findings establish LJP as a promising natural agent for the prevention of acute lung injury.

## 2. Materials and Methods

### 2.1. Materials and Reagents

LJP powder was prepared using our previous method [20]. In brief, the dried *Lonicerae japonicae flos* (Batch No. 220601) was purchased from Shandong Baiweitang Co., Ltd. (Jinan, China), crushed and sieved, and then extracted with a 10-fold volume (*v*/*w*) of 40% (*v*/*v*) ethanol under reflux for 2 h. After concentration, the supernatant was defatted by petroleum ether extraction, freeze-dried, and stored for further use. LJP was finally isolated by pH-zone-refining counter-current chromatography. It mainly contains neochlorogenic acid (1.92%), chlorogenic acid (59.65%), cryptochlorogenic acid (5.37%), isochlorogenic acid B (3.80%), isochlorogenic acid A (19.66%), and isochlorogenic acid C (9.60%) of the total polyphenol fraction, with an overall purity of 83.29% (Figures S1 and S2, Tables S1 and S2). Lipopolysaccharide, liproxstatin-1, and dexamethasone were purchased from Sigma-Aldrich Trading (Shanghai, China), Macklin Biochemical (Shanghai, China) and Aladdin Biochemical Technology (Shanghai, China), respectively. Antibody information is shown in Table S3.

### 2.2. Animals and Treatment

Eighty-four male C57BL/6J mice (8 weeks old) were randomly divided into seven groups (*n* = 12) using a computer-generated random number table: saline control group (CON), model group (MOD), dexamethasone group (DXM, 5 mg/kg bw/d), liproxstatin-1 group (LIP, 10 mg/kg bw/d), and high/medium/low-dose LJP groups (200/100/50 mg/kg bw/d). Four mice were kept per cage in an SPF environment (22 ± 2 °C, 12 h light/dark cycle) with free access to food and water. Experiments started after 7 days of acclimatization. The ALI mouse model and dosing scheme were established with some modifications based on previous reports [21]. All groups were pretreated for 7 days, with dexamethasone and liproxstatin-1 group treatments administered via intraperitoneal injection and other groups’ by gavage [22,23]. On the 7th day, all groups except the CON group received intratracheal instillation of LPS (5 mg/kg) to establish the ALI mouse model [24]. Body weights and dietary intake were recorded daily during the experiment. All animal experiments were conducted at Shandong Institute of Traditional Chinese Medicine (License No.: SYXK (Lu) 20230004), and the study protocol underwent review and received approval from the Institute’s Animal Ethics Committee (Approval No.: SDZYY20240116001).

### 2.3. Experimental Sample Collection and Processing

**Figure 1:**
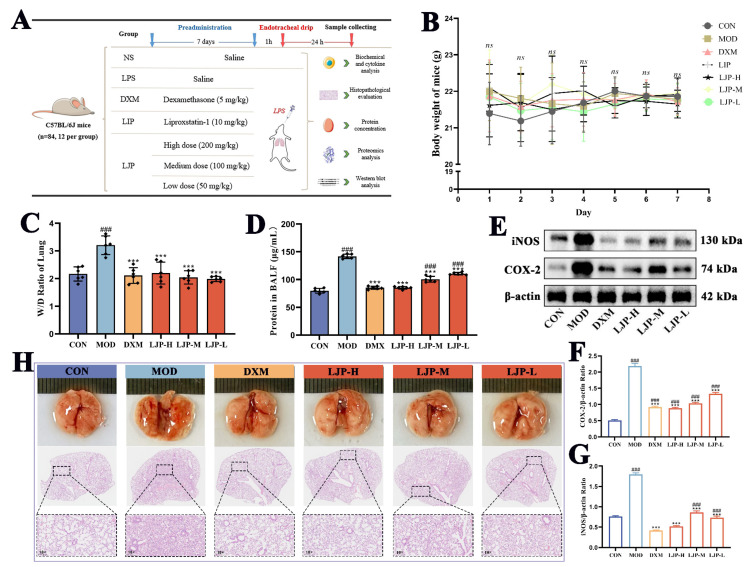
LJP mitigated LPS-induced pulmonary edema and histopathological damage in ALI mice (*n* = 12). (**A**) Experimental animals and grouping. (**B**) Daily body weight of mice. (**C**) Lung tissue W/D ratio. (**D**) Leakage protein in BALF. (**E**) Western blot analysis of inflammatory mediators. (**F**,**G**) The expression levels of COX-2 and iNOS. (**H**) The histopathological damage of lung tissue was observed by HE staining. (×10, scale bar = 40 μm). ^###^ *p* < 0.001 (vs. CON group); *** *p* < 0.001 (vs. MOD group).

For each group of 12 mice, six were randomly assigned to harvest bronchoalveolar lavage fluid (BALF), and the remaining six were processed for lung tissue collection. ALI modeling was performed 1 h after the final administration on day 7 (Figure 1A). One day after modeling, serum, lung tissues, and fresh feces were collected for further analysis [25]. BALF collection: The trachea was exposed via neck dissection; a small incision was made for blunt-tip catheter insertion. 1 mL of PBS was slowly infused and then withdrawn to collect lavage fluid [26]. The wet weight of the right lung was measured first, followed by drying at 70 °C to constant weight for calculation of the wet/dry ratio (W/D). All collected animal data were included in the final analysis without exclusions. During data analysis, group identities were coded, and the analyst remained blinded until all analyses were completed.

### 2.4. LJP-Derived FMT Trial

C57BL/6J mice (male, 6 weeks old) as the donor mice were randomly divided into two groups (*n* = 6): control donor and LJP-derived donor. The control donor group was pretreated with PBS by gavage, while the LJP donor group was orally administered 200 mg/kg/d of LJP for 7 days. A total of 1 g of fresh feces was collected from each donor mouse, then mixed, homogenized, and suspended in 10 mL of PBS containing 10% glycerol for FMT as previously described. Finally, 1 mL of the bacterial suspension was transferred to a test tube and frozen at −80 °C for further experiments.

Next, C57BL/6J receptor mice (male, 8 weeks old) were selected for the FMT experiment and randomly divided into 3 groups (*n* = 6): MOD group (PBS), FMT-CON group (intragastric administration of control donor-derived fecal microbiota), and FMT-LJP group (intragastric administration of LJP donor-derived fecal microbiota). Meanwhile, all mice were orally administered fecal microbiota or PBS for 7 consecutive days. On the 8th day, LPS (5 mg/kg) was administered to all groups to induce acute lung injury. One day later, after anesthesia and orbital venous blood collection, the mice were euthanized via cervical dislocation, after which lung, fresh fecal and colon tissue samples were harvested for testing.

### 2.5. Biochemical and Cytokine Analysis

Levels of SOD, ROS, Fe^2+^, GSH, GSSG, and MDA were determined with commercially available assay kits (Jiancheng Biology Technology Co., Ltd., Nanjing, China). Tissue homogenates were analyzed for IL-6, IL-1β, and TNF-α levels employing ELISA kits (Neobioscience Co., Ltd., Beijing, China).

### 2.6. Lung Histopathological Analysis

Following 4% paraformaldehyde fixation, lung tissues underwent paraffin embedding, microtome sectioning, and histological analysis. Histopathological alterations in the lung were evaluated using H&E staining under a light microscope. The images were captured under an Olympus VS200 slide scanner and analyzed using ImageJ software (ImageJ/Fiji 2.1).

### 2.7. Immunofluorescence Analysis

Formalin-fixed paraffin-embedded (FFPE) sections underwent xylene-based deparaffinization and alcohol-gradient rehydration. Sequential multiplex immunohistochemistry employed iterative cycles of protein blocking with 1% BSA, primary antibody incubation, species-specific HRP-conjugated secondary antibody (Akoya Biosciences, Marlborough, MA, USA), and Opal fluorophore (1:100 in Akoya diluent) application with tyramide signal amplification. Antibody stripping via microwave antigen retrieval preceded subsequent cycles. Tissue sections underwent spectral DAPI counterstaining (Akoya Biosciences), and sections were mounted with anti-fade medium (Abcam ab104135). Images were captured at ×200 magnification using the PANNORAMIC SCAN II system (3Dhistech).

### 2.8. Proteomic Analysis

Frozen samples were homogenized in protein lysis buffer using high-throughput tissue grinding, incubated on ice for 30 min with periodic vortexing, and centrifuged. BCA assay was utilized for the measurement of supernatant protein concentration. For digestion, proteins (100 µg) were resuspended in TEAB (100 mM), reduced with TCEP (10 mM, 37 °C, 60 min), alkylated with IAM (40 mM, RT, dark, 40 min), centrifuged and digested with trypsin. Peptides were vacuum dried, reconstituted in 0.1% TFA, desalted (HLB), and subsequently quantified (Thermo Fisher Peptide Quantification Kit, Waltham, MA, USA). Equal peptide amounts were analyzed via DIA-MS on a VanquishNeo-Orbitrap Astral system (Thermo).

### 2.9. Western Blot Analysis

Lung tissue (20 mg) was lysed in RIPA buffer and centrifuged (10,000 *g*, 15 min, 4 °C). The samples were measured (BCA assay) and mixed with 5× SDS-PAGE buffer, centrifuged, and denatured (100 °C, 5 min). Gels: 10% separating gel (solidified 15 min), ethanol-sealed; stacking gel with comb (RT, 15 min). Denatured samples (95 °C, 5 min) and marker were electrophoresed at 80 V (30 min) then 120 V (60 min). We transferred them to methanol-activated PVDF membrane (400 mA, 50 min, 4 °C) in transfer buffer. Assays were performed after blocking and antibody incubation. Immunoreactivity was visualized via ECL chemiluminescence and signals were imaged and quantified using ImageJ.

### 2.10. Gut Microbiota Analyses

The intestinal microbiota was analyzed using high-throughput 16S rRNA sequencing technology. PCR amplification was performed according to the original method [27]. The V3-V4 region of the 16S rRNA gene was amplified with barcoded primers 338F (5′-ACTCCTACGGGAGGCAGCAG-3′) and 806R (5′-GGACTACHVGGGTWTCTAAT-3′). PCR products were confirmed via agarose gel electrophoresis, purified using the Universal DNA Purification Recovery Kit (Tiangen Biotech, Beijing, China), and quantified with Q-PCR (Bio-Rad T100, Hercules, CA, USA). Sequencing was performed on an Illumina NovaSeq 6000 platform (Illumina, San Diego, CA, USA).

### 2.11. Measurement of Short-Chain Fatty Acids

A total of 20 mg of fecal samples was homogenized in 800 μL 0.5% H_3_PO_4_ through cryomilling, followed by sonication and centrifugation. The supernatant (200 μL) underwent *n*-butanol extraction (1:1 *v*/*v*) and was vortex-mixed, sonicated (10 min), and centrifuged. The organic phase was analyzed by GC-MS (Agilent 8890B-5977B, Santa Clara, CA, USA).

### 2.12. Statistical Analysis

Data were expressed as means ± standard deviation (SD). All bar plots were generated using GraphPad Prism 8.0 (GraphPad Software, San Diego, CA, USA). Statistical differences were determined by one-way analysis of variance (ANOVA), along with Tukey’s test at *p* < 0.05, *p* < 0.01 or *p* < 0.001.

## 3. Results

### 3.1. LJP Mitigated LPS-Induced Pulmonary Edema and Pathological Changes in ALI Mice

Prior to LPS administration, the body weights of mice in each group remained stable within the range of 21 ± 0.5 g (Figure 1B). The lung W/D ratio in the MOD group reached 3.21 (Figure 1C), and the concentration of leaked proteins in BALF reached 141.82 μg/mL (Figure 1D), which were approximately 1.47-fold and 1.78-fold higher than those in the CON group, respectively. Additionally, expressions of pro-inflammatory mediators (iNOS and COX-2) were remarkably increased in the MOD group (*p* < 0.001) (Figure 1E–G), indicative of severe local and systemic inflammatory response, with concomitant damage to alveolar epithelial cells [28]. LPS induced significant lung injury, characterized by edema, excessive mucus production, and widespread hemorrhage. Histopathological examination via HE staining revealed pronounced interstitial thickening, accompanied by inflammatory cell infiltration and alveolar structure destruction in the MOD group (Figure 1H). These findings confirmed the successful establishment of the ALI mouse model [29]. However, the DXM and LJP groups all significantly reduced the lung W/D ratio and concentration of leaked proteins in BALF (*p* < 0.001). Meanwhile, LJP significantly downregulated the expression of iNOS and COX-2 (*p* < 0.001), and inhibited the inflammatory response caused by pro-inflammatory mediators.

### 3.2. Proteomic Changes After LJP Intervention

**Figure 2:**
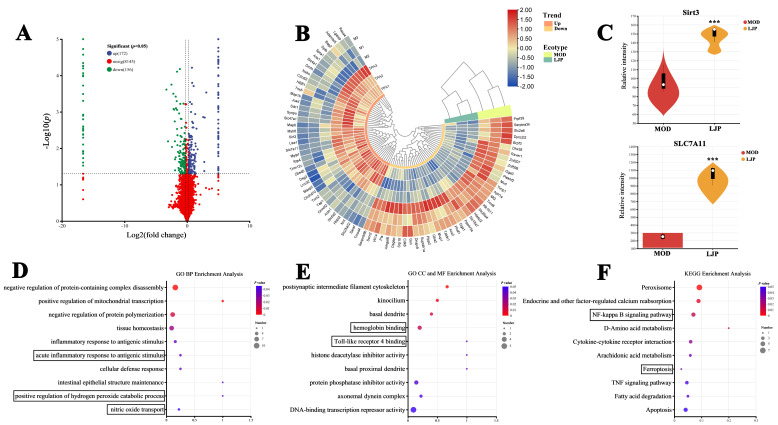
Proteomic analysis of the MOD and LJP groups (*n* = 3). (**A**) Volcano plot of the MOD and LJP groups’ protein. (**B**) Heat map of the top 80 differentially expressed proteins (DEPs) in 328 drawn by TBtools (v2.154). (**C**) The expression levels of Sirt3 and SLC7A11, which were statistically significant between the MOD and LJP groups. (**D**) GO BP enrichment analysis of 328 DEPs. (**E**) GO CC and MF enrichment analysis of 328 DEPs. (**F**) KEGG pathway analysis of 328 DEPs. *** *p* < 0.001.

DIA-based proteomics quantitative analysis technology was used to analyze lung tissues of MOD and LJP groups [30]. As shown in Figure 2A, a total of 8473 proteins were identified, with 1558 proteins subjected to quantitative analysis. Using the above criteria of fold-change thresholds of 1.2 (upregulation) and 0.83 (downregulation), 328 differentially expressed proteins (DEPs) were identified (*p* < 0.05), of which 156 were downregulated and 172 were upregulated. The top 80 DEPs were displayed in Figure 2B. Additionally, GO and KEGG enrichment analyses were conducted to collectively characterize the 328 DEPs (Figure 2D–F). The vast majority of GO biological process (BP) terms were related to inflammatory responses and oxidative stress, such as antigenic-induced acute inflammatory reaction, positive regulation of hydrogen peroxide catabolic process, and nitric oxide transport. Notably, the GO cellular component (CC) and molecular function (MF) categories highlighted key terms including hemoglobin interaction and Toll-like receptor 4 (TLR4) binding, both of which are implicated in inflammatory response and oxidative stress [31].

KEGG pathway analysis revealed that ferroptosis and NF-κB pathways were closely related to the aforementioned GO enrichment analysis. When cells were stimulated by LPS, the Toll-like receptor 4 binding mediated NF-κB pathway was activated, which promoted the production of pro-inflammatory factors such as IL-6 and TNF-α, subsequently triggering oxidative stress, lipid peroxidation, and ferroptosis [32]. Regarding ferroptosis, Sirt3 and SLC7A11 proteins detected in DEPs were identified as the key regulators of ferroptosis (Figure 2C) [33]. In conclusion, proteomic analysis revealed that LJP modulated protein expression in ALI mice mainly through pathways associated with inflammation, oxidative stress, lipid peroxidation, and ferroptosis, which were further validated by the subsequent experiments.

### 3.3. LJP Mitigated TLR4/MyD88/NF-κB Pathway Activation

**Figure 3:**
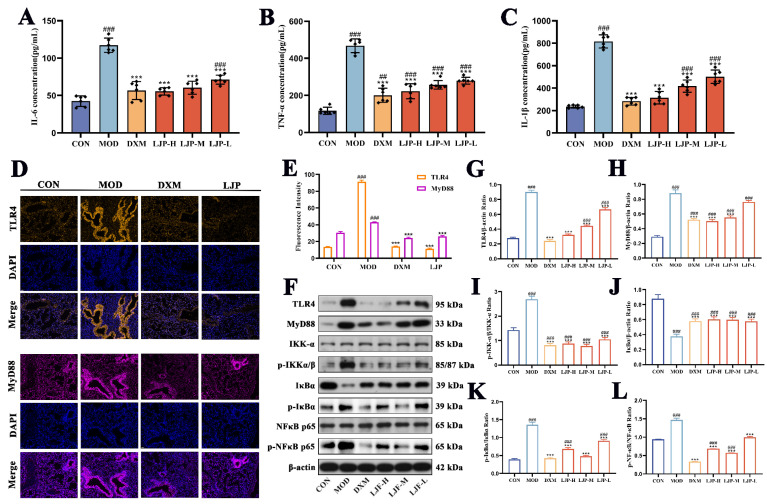
LJP ameliorated lung inflammation in ALI mice by mitigating TLR4/MyD88/NF-κB pathway activation. (**A**–**C**) Levels of IL-6, TNF-α and IL-1β in lung tissues. (**D**) Immunofluorescence analysis of TLR4 and MyD88 in lung tissue. (**E**) The fluorescence intensity of the protein measured by ImageJ. (**F**) Western blot analysis of TLR4/MyD88/NF-κB signaling pathway. The expression levels of (**G**) TLR4, (**H**) MyD88, (**I**) p-IKKα/β, (**J**) IĸBα, (**K**) p-IĸBα, (**L**) p-NF-κB (p65). ^##^ *p* < 0.01, ^###^ *p* < 0.001 (vs. CON group); *** *p* < 0.001 (vs. MOD group).

The activation of the TLR4/MyD88/NF-κB pathway promoted the release of inflammatory factors. Compared with the MOD group, pretreatment with a high dose of LJP markedly reduced the expression levels of TNF-α, IL-6, and IL-1β to 61.7 pg/mL, 245.3 pg/mL, and 502.3 pg/mL, respectively (Figure 3A–C). In general, NF-κB binds to the IκBα protein and resides in the cytoplasm [34]. Upon stimulation, IκBα undergoes phosphorylation and subsequent degradation, leading to the nuclear translocation of NF-κB (p65), which subsequently drives the transcription of inflammatory mediators [35].

The immunofluorescence analysis of TLR4 and MyD88 proteins showed that the fluorescence values in the MOD group were 6.67-fold and 1.40-fold higher than those in the CON group (Figure 3D,E), and the fluorescence value decreased significantly in the LJP group (*p* < 0.001). Western blot analysis further confirmed that dexamethasone (DXM) and LJP pretreatment exhibited significant decreases in TLR4, MyD88, p-IKKα/β, p-IκBα, and p-NF-κB (p65) protein expression (*p* < 0.001) (Figure 3F–L). Collectively, these results preliminarily showed that LJP could ameliorate lung inflammation in ALI mice by inhibiting the activation of the TLR4/MyD88/NF-κB pathway.

### 3.4. LJP Activated Nrf2/HO-1 and Sirt3/Nrf2/GPX4 Pathways

**Figure 4:**
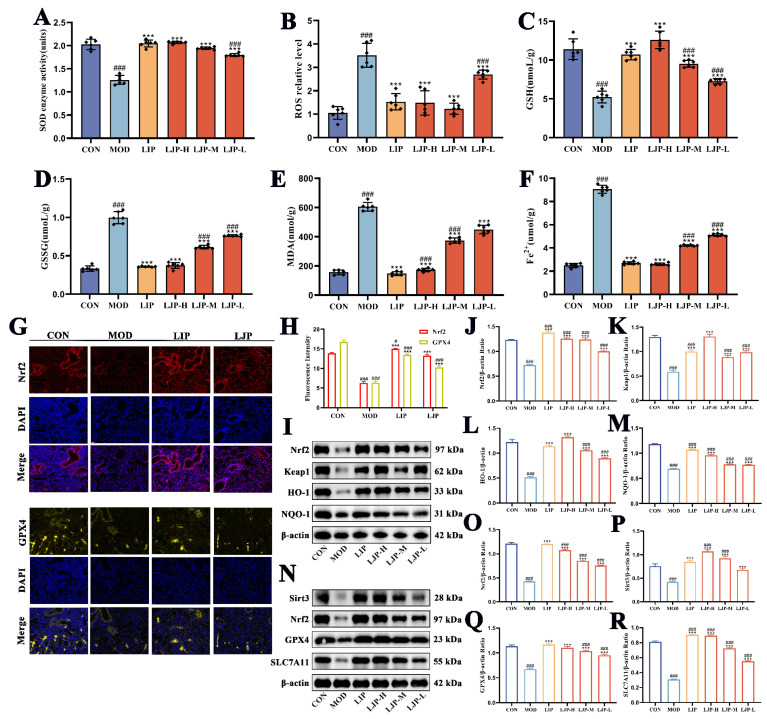
LJP ameliorated lung oxidative stress, lipid peroxidation and ferroptosis in ALI mice by activating Nrf2/HO-1 and Sirt3/Nrf2/GPX4 pathways. (**A**–**F**) The levels of SOD, ROS, GSH, GSSG, MDA and Fe^2+^ ions in lung tissue. (**G**) Immunofluorescence analysis of Nrf2 and GPX4 in lung tissue. (**H**) ImageJ measures the fluorescence intensity of the protein Nrf2 and GPX4. (**I**) Western blot analysis of Nrf2/HO-1 pathway. The expression levels of (**J**) Nrf2, (**K**) Keap1, (**L**) HO-1, (**M**) NQO-1. (**N**) Western blot analysis of Sirt3/Nrf2/GPX4 pathways. The expression levels of (**O**) Sirt3, (**P**) Nrf2, (**Q**) GPX4, (**R**) SLC7A11. ^#^ *p* < 0.05, ^###^ *p* < 0.001 (vs. CON group); *** *p* < 0.001 (vs. MOD group).

To validate LJP’s involvement in inhibiting ferroptosis, the relevant physiological indicators in the lung tissue were analyzed, using liproxstatin-1 (LIP) as the positive control group. In contrast, dexamethasone (DXM), a broad-spectrum anti-inflammatory agent lacking specific validated regulatory activity toward the ferroptosis pathway, was excluded from these analyses. The results show that the levels of SOD and GSH in the lung tissues of both LIP and LJP-H groups were remarkably increased (*p* < 0.001), which correlated with reduced accumulation of ROS, MDA, and Fe^2+^ [36]. In particular, the SOD and GSH expression levels in the LJP-H group were 1.64-fold and 2.40-fold (*p* < 0.001) higher than those in the MOD group, respectively (Figure 4A,C). In contrast, ROS, GSSG, MDA, and Fe^2+^ expression levels in the LJP-H group were 2.37-fold, 2.67-fold, 3.50-fold, and 3.48-fold (*p* < 0.001) lower than those in the MOD group, respectively (Figure 4B,D–F). To further assess the activation of key proteins involved in anti-ferroptosis pathways, immunofluorescence and Western blot analysis were performed to evaluate key proteins Nrf2 and GPX4. The immunofluorescence analysis showed that the fluorescence values in the LJP-H group were 2.11-fold and 1.60-fold higher than those in the MOD group (Figure 4G,H). Compared with the MOD group, Western blot analysis indicated that Nrf2, Keap1, HO-1, NQO-1, Sirt3, GPX4, and SLC7A11 expression levels were markedly increased (*p* < 0.001) in the LIP and LJP groups (Figure 4I–R). These findings indicated that LJP could ameliorate lung oxidative stress, lipid peroxidation and ferroptosis in ALI mice by activating the Nrf2/HO-1 and Sirt3/Nrf2/GPX4 pathways [37], thereby exerting a protective effect against lung injury induced by ALI.

### 3.5. Effect of LJP on Gut Microbiota and SCFA Content in ALI Mice

**Figure 5:**
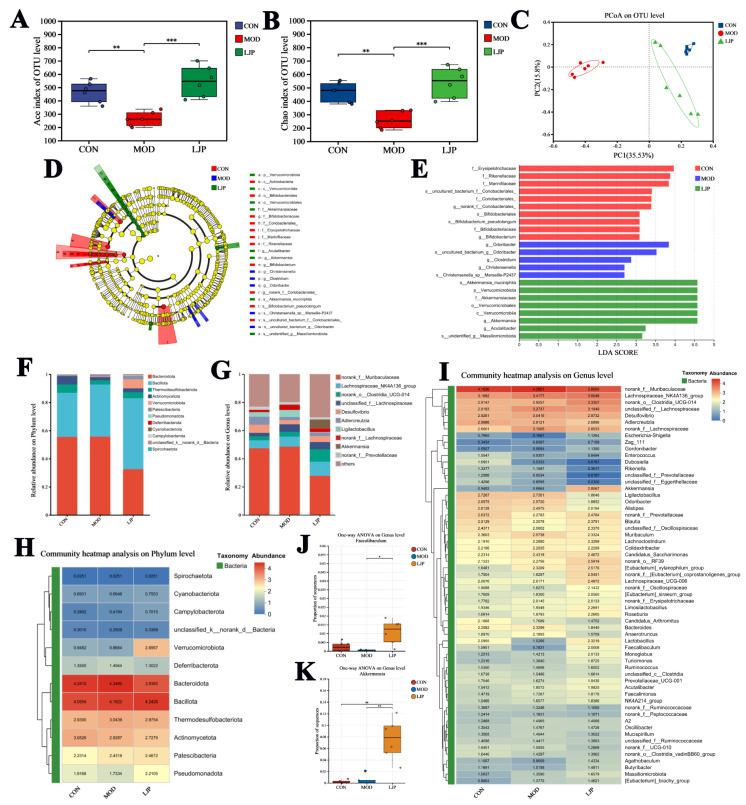
Effect of LJP on gut microbiota in ALI mice. (**A**) Ace index of OTU level. (**B**) Chao index of OTU level. (**C**) PCoA on OTU level. (**D**,**E**) Differential species analysis, Cladogram plots, Lefse analysis LDA value >4. (**F**,**G**) Bacterial taxonomic profiling at the phylum and genus level. (**H**,**I**) Relative abundance at top 12 phyla and top 60 genera shown as a heat map. (**J**,**K**) The expression levels of *Faecalibaculum* and *Akkermansia,* which were statistically significant between the MOD and LJP groups. Data are expressed as the mean ± SD, *n* = 6. * *p* < 0.05, ** *p* < 0.01, *** *p* < 0.001.

The MOD group exhibited a significant reduction in α-diversity indices (Ace and Chao) compared to the CON group (*p* < 0.01), reflecting diminished richness in the gut microbial communities of ALI mice [38]. LJP intervention significantly enhanced α-diversity, reflecting enhanced species richness within the gut microbiota (Figure 5A,B). Principal coordinate analysis (PCoA) revealed clear separation among groups based on microbial community composition (Figure 5C). Furthermore, shifts in community structure were assessed by profiling taxonomic composition at both the phylum and genus levels. Linear discriminant analysis Effect Size (LEfSe) was employed to identify differentially abundant biomarkers, revealing that 23 OTUs (LDA > 4) exhibited significant differences spanning phylum to genus levels, suggesting distinct microbial community structures between groups (Figure 5D,E). Changes in the microbial community were explored at the level of phylum and genera via heatmap presentation, showing the relative abundance of top 12 phyla and top 60 genera with the highest abundance in each group (Figure 5H,I). The microbial communities at the genus level in the CON, MOD and LJP groups were mainly composed of *Bacillota* and *Bacteroidota* (Figure 5F). At the phylum level, the relative abundance of *Verrucomicrobiota* and *Bacillota* was more abundant in the LJP group compared to the CON and MOD groups. At the genus level, *norank_f_Muribaculaceae* and *Lachnospiraceae_NK4A136_*groups were the main bacterial communities across the microbial communities of CON, MOD, and LJP groups (Figure 5G). Notably, LJP pretreatment markedly elevated the relative abundance of *Faecalibaculum* and *Akkermansia* as compared to the CON and MOD groups (Figure 5J,K), and decreased the relative abundance of *norank_f_Muribaculaceae*, *Enterococcus*, *Dubosiella*, *Rikenella* and others.

**Figure 6:**
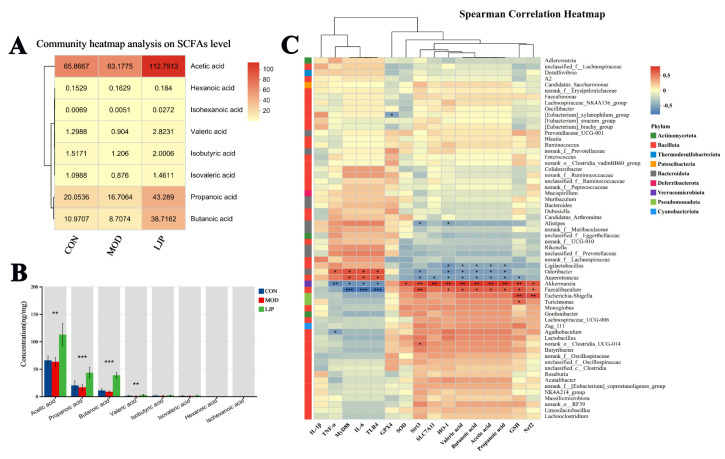
Effect of LJP on SCFA content in ALI mice. (**A**,**B**) Concentrations of short-chain fatty acids (SCFAs) in fresh feces. (**C**) Heatmap of Spearman’s rank correlations between ALI-related parameters and genus-level alterations in the gut microbiota. * *p* < 0.05, ** *p* < 0.01, *** *p* < 0.001.

In summary, relative to the MOD group, LJP treatment markedly reshaped gut microbiota composition and elevated the abundances of *Faecalibaculum* (*p* < 0.05) and *Akkermansia* (*p* < 0.01), which are classified under phyla *Bacillota* and *Verrucomicrobiota*, respectively. Consistent with this, phylum-level analysis revealed elevated relative abundances of *Bacillota* and *Verrucomicrobiota*, corroborating the genus-level findings and indicating a coherent taxonomic shift across classification levels. Furthermore, the production of short-chain fatty acids, including acetic acid, propionic acid and butanoic acid, was markedly elevated in the LJP group (Figure 6A,B). Correlation analysis was performed to elucidate the relationships between ALI-related parameters and genus-level alterations in the gut microbiota. The results were visualized via a heatmap (Figure 6C). The findings indicated that *Faecalibaculum* and *Akkermansia* exhibited positive correlations with SCFAs (acetic acid, propionic acid and butanoic acid), antioxidant-related parameters (GSH, SOD, Nrf2, and HO-1), and ferroptosis-inhibiting proteins (Sirt3 and SLC7A11), while being negatively correlated with inflammation-related parameters (IL-6, TNF-α, IL-1β, TLR4, and MyD88). The results suggested a potential link between LJP-induced gut microbiota remodeling and the attenuation of ALI.

### 3.6. FMT with LJP-Derived Gut Microbiota Improved the LPS-Induced ALI in Mice

**Figure 7:**
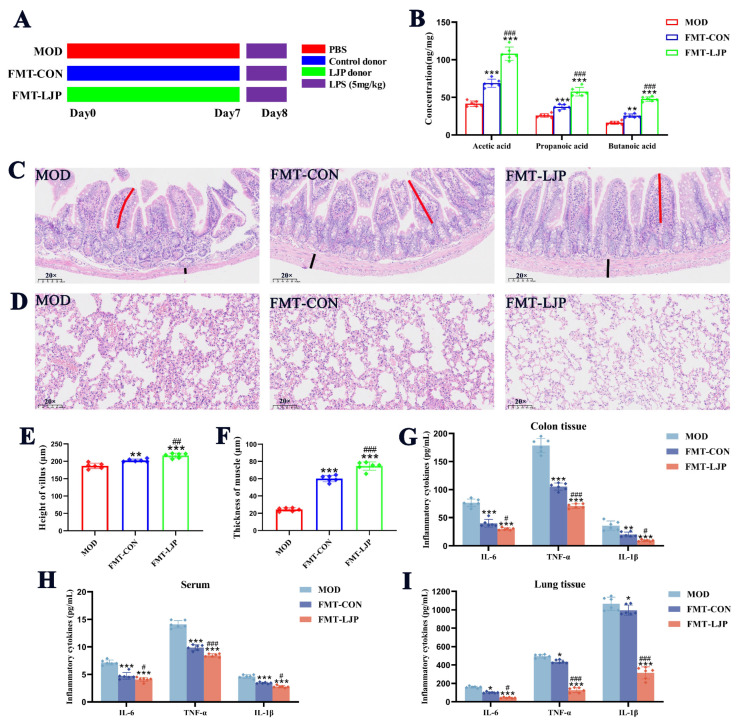
FMT with LJP-derived gut microbiota improved the LPS-induced ALI in mice (*n* = 6). (**A**) Schematic diagram of the experimental design. (**B**) Concentrations of short-chain fatty acids (SCFAs) in intestinal tract contents. Representative images of H&E staining (×20, scale bar = 20 μm) in (**C**) colon tissues and (**D**) lung tissues. (**E**) Height of villus and (**F**) thickness of muscle in the colon tissues. Levels of IL-6, TNF-α, and IL-1β in (**G**) colon tissues, (**H**) serum and (**I**) lung tissues. MOD: treated with LPS as the model group; FMT-CON: treated with LPS with FMT from the control donor; and FMT-LJP: treated with LPS with FMT from the LJP donor. Data are expressed as means ± SD (*n* = 6). ^#^ *p* < 0.05, ^##^ *p* < 0.01, ^###^ *p* < 0.001 (vs. FMT-CON group); * *p* < 0.05, ** *p* < 0.01, *** *p* < 0.001 (vs. MOD group).

The effectiveness of LJP-derived fecal microbiota was validated based on alleviating ALI symptoms in mice (Figure 7A). As depicted in Figure 7B, compared to the FMT-CON and MOD groups, the significant elevations in acetic acid, propanoic acid, and butanoic acid were observed in the FMT-LJP group (all *p* < 0.01). Consistent with these findings, the HE staining results showed that colonic sections from the FMT-LJP group showed a complete and clear villus structure, in comparison with both FMT-CON and MOD groups (*p* < 0.01) (Figure 7C,E). Moreover, the villus height and muscle thickness in FMT-LJP group were notably greater than those in both FMT-CON and MOD groups (*p* < 0.01) (Figure 7C,F). Meanwhile, lung tissues from the FMT-LJP group showed significant improvement in pulmonary interstitial thickening and inflammatory cell infiltration (Figure 7D). Notably, pro-inflammatory factor levels in the serum, colon, and lung tissue were significantly lower in the FMT-LJP group than those in the MOD group (all *p* < 0.05) (Figure 7G–I). The results demonstrated that LJP reshaped the gut microbiota, increased SCFA production, and was associated with alleviation of lung tissue injury and intestinal barrier damage in ALI mice.

## 4. Discussion

Lung inflammation induces the secretion of pro-inflammatory factors, further driving oxidative stress, lipid peroxidation and ferroptosis [39]. Meanwhile, intestinal inflammation damages the intestinal barrier by altering the microbiota, reducing beneficial bacteria and impairing gut function [40,41]. Damaged intestinal tissue releases inflammatory factors into the circulation, which in turn aggravates pulmonary inflammation [42]. Most existing gut-targeted therapeutic studies focus on macromolecules such as plant-derived polysaccharides and dietary fibers [43], whereas small-molecule compounds have received far less attention for gut regulation. Among these, polyphenols are increasingly recognized as important regulators of intestinal and pulmonary anti-inflammatory effects [44]. Meanwhile, polyphenols were associated with increased abundance of beneficial gut bacteria and SCFAs [45], which in turn maintained the intestinal barrier and reduced the concentration of inflammatory factors in the bloodstream [46], thereby mitigating lung injury progression. For instance, *Aronia melanocarpa* polyphenols enhanced intestinal barrier function, thereby decreasing serum levels of LPS and modulating the gut microbiota in LPS-induced liver disease [47]. *Rosa roxburghii* Tratt polyphenols confer protection against ALI, primarily through intestinal barrier restoration and SCFA enhancement. These effects may contribute to remodeling the population of *Akkermansia muciniphila* and normalizing dysregulated endogenous metabolites [48]. Overproduction of pro-inflammatory cytokines damages alveolar epithelial cells and disrupts the intestinal barrier [49]. Therefore, the ability to inhibit lung inflammation is a key factor when evaluating potential drugs for the prevention or treatment of ALI, thereby inhibiting the inflammation-related ferroptosis.

Given the satisfactory protective efficacy of LJP against ALI, we further investigated its underlying molecular mechanisms in lung tissue. Further exploration showed that LJP inhibited the TLR4/MyD88/NF-κB pathway to suppress lung inflammation and reduce inflammatory factor release. Upon activation of TLR4 by lipopolysaccharides, the adaptor protein MyD88 initiates signaling that results in phosphorylation of IKKα/β and IκBα [50]. This process promotes nuclear translocation of NF-κB p65 and subsequently upregulates inflammatory cytokine expression [51]. During infectious and inflammatory states, excessive accumulation of ROS can lead to an imbalance in cellular antioxidant defenses [52]. Excess ROS damages lipids, proteins and DNA, elevates lipid peroxidation in pulmonary epithelial and endothelial cells, and ultimately promotes ferroptosis [53]. It is a stepwise pathological process that aggravates lung damage. Meanwhile, LJP activated Nrf2/HO-1 and Sirt3/Nrf2/GPX4 pathways, leading to remarkable biochemical changes including the increased levels of SOD and GSH, and the decreased accumulation of ROS, GSSG, MDA, and Fe^2+^, effectively inhibiting ferroptosis. Sirt3 is a mitochondrial deacetylase that regulates the function of multiple mitochondrial proteins through deacetylation, playing a crucial role in maintaining cellular energy metabolism, combating oxidative stress, and delaying aging [54]. Under external stimulation, Sirt3 deacetylates Nrf2 to enhance its activity. Nrf2 then dissociates from Keap1, translocating into the nucleus and upregulating the expression of antioxidant proteins such as HO-1, NQO-1, and GPX4 [55,56,57]. This enhanced antioxidant response effectively scavenges LPS-induced excess ROS and reduces MDA levels, thereby mitigating oxidative damage [58]. Additionally, SLC7A11 is a key transporter for cysteine, an essential precursor for GSH. Under the combined action of GPX4, it effectively inhibits the accumulation of lipid peroxide GSSG, thereby indirectly suppressing lipid peroxidation and ferroptosis [59].

To investigate whether gut microbiota was associated with the protective effects of LJP, we conducted fecal microbiota transplantation (FMT) assays. FMT serves as an effective therapeutic approach for ameliorating ALI by regulating the intestinal flora dysbiosis through the transplantation of functional live bacteria. Transplantation of LJP-derived microbiota significantly mitigated lung tissue injury and intestinal barrier damage in ALI mice. These findings indicated that the protective effects of LJP against ALI were associated with gut microbiota remodeling and elevated SCFA production. Additionally, this study has several limitations. The absence of a healthy control in the FMT trial limits the evaluation of the protective effects of FMT-LJP in ALI mice. Since only six predominant phenolic acids are quantified to determine LJP purity, additional polyphenolic components such as flavonoids require further analysis, which may contribute synergistically to alleviating acute lung injury. In addition, blood and liver biomarkers critical for assessing systemic toxicity remained untested. Further preclinical and clinical studies are required to fully evaluate the toxicological profile of LJP.

## 5. Conclusions

In conclusion, this study demonstrated that LJP exerted a protective effect against ALI by attenuating inflammation-related ferroptosis and gut microbiota dysbiosis. LJP substantially increased the abundance of beneficial gut bacteria (*Akkermansia muciniphila* and *Faecalibaculum*) and the production of SCFAs. Furthermore, in the FMT validation experiment, LJP-derived gut microbiota significantly alleviated lung tissue injury and intestinal barrier damage in ALI mice. Our findings suggest that LJP shows promising potential for preventing acute lung injury in mice.

## Data Availability

Data is contained within the article or Supplementary Material. Accession numbers for proteomics and 16S rRNA sequencing data are available in public repositories: iProX (IPX0017757000) and NCBI BioProject (PRJNA1478188).

## Supplementary Materials

The following supporting information can be downloaded at: https://www.mdpi.com/article/10.3390/nu18132048/s1, Figure S1: Chemical structures of the main constituents in LJP: (A) neochlorogenic acid, (B) chlorogenic acid, (C) cryptochlorogenic acid, (D) isochlorogenic acid B, (E) isochlorogenic acid A, and (F) isochlorogenic acid C. Figure S2: HPLC chromatograms of (A) standard samples and (B) LJP. (1. neochlorogenic acid, 2. chlorogen-ic acid, 3. cryptochlorogenic acid, 4. isochlorogenic acid B, 5. isochlorogenic acid A, 6. isochlorogenic acid C). Table S1: Regression equations and linear ranges of LJP. Table S2: Spike recoveries of LJP. Table S3: Antibody information.
