# Supplementary material for: Lonicerae japonicae flos Polyphenols Attenuate Inflammation-Related Ferroptosis and Gut Microbiota Dysbiosis in LPS-Induced Acute Lung Injury in Mice
Source: Nutrients. 2026 Jun 23;18(13):2048. doi: 10.3390/nu18132048 (PMC13363504; doi:10.3390/nu18132048)
Supplement: Supplementary file 1 [file nutrients-18-02048-s001.zip › nutrients-4373869-supplementary.pdf]

Supplementary material

Chemical structures of the main constituents in LJP

The chemical structures of neochlorogenic acid, chlorogenic acid, cryptochlorogenic acid, isochlorogenic acid B, isochlorogenic acid A, and isochlorogenic acid C have been presented in Figure S1.

LJP powder sample analysis

1.1. Preparation of test solution

The LJP sample was precisely weighed and added with methanol at a constant volume of 10.0 mg/mL, and then passed through a 0.22 μm organic filter membrane.

1.2. Preparation of standard sample solution

The standard components of neochlorogenic acid, chlorogenic acid, cryptochlorogenic acid, isochlorogenic acid B, isochlorogenic acid A and isochlorogenic acid C were precisely weighed and dissolved in methanol solution for constant volume. The mass concentration of each component was 6.0 mg/mL of chlorogenic acid, and 2.0 mg/mL of neochlorogenic acid, cryptochlorogenic acid, isochlorogenic acid B, isochlorogenic acid A and isochlorogenic acid C. The above mixed control solution was further diluted into a known concentration of mixed control solution and stored at 4 °C. The HPLC chromatograms of mixed standard sample and LJP sample is shown in Figure S2.

1.3. Chromatographic conditions

Agilent 1260 HPLC was performed on a Waters C18 (250 mm×4.6 mm, 5 μm) column. Mobile phase: acetonitrile-0.2% formic acid water, gradient elution: 0-10 min, 8%-10% acetonitrile; 10 ~ 20 min, 10% ~ 15% acetonitrile; 20-30 min, 15% acetonitrile; 30-40 min, 15%-25% acetonitrile; 40 ~ 50 min, 25% ~ 30% acetonitrile; 50-51 min, 30%-100% acetonitrile; 51-56 min, 100% acetonitrile; 56-57 min, 100%-8% acetonitrile; 57-62 min, 8% acetonitrile; The volume flow was 1.0 mg/min; The injection volume was 2.0 μL. The detection wavelength was 327 nm.

1.4. Methodology investigation

Precision test: The known concentration of the mixed reference solution was detected according to the chromatographic conditions of item "1.3" above, the sample was injected for 6 times continuously, and the

peak areas of the chromatographic peaks of the 6 reference materials were recorded. The relative standard deviation (RSD) of the calculated peak area was 0.15% ~ 3.12%, indicating that the instrument had good precision.

Repeatability test: Six sample solutions were prepared and determined according to the above chromatographic conditions under item "1.3", and the mass fractions of six reference materials were calculated. The RSD of the mass fractions of each reference substance was 0.69% ~ 3.69%, indicating that the method had good repeatability.

Stability test: According to the chromatographic conditions of item "1.3" above, the test solution was injected at 0, 2, 4, 8, 12 and 24 hours, respectively, and the chromatographic peak areas of the six control substances were recorded. The results showed that the RSD value of the peak area of each reference substance ranged from 0.31% to 1.72%, which proved that the test substance solution showed good stability within 24 hours.

Linear relationship investigation: each reference solution was accurately measured and diluted with methanol gradient to determine the appropriate concentration used to construct the standard curve. The determination was performed according to the chromatographic conditions of item "1.3" above. The standard curve was drawn with the mass concentration of the reference as the abscissa (x) and the peak area (mV·s) as the ordinate (y), and linear regression analysis was performed. Finally, the regression equations, correlation coefficients and linear ranges of the six components were obtained, and the data are shown in Table S1.

Spike recovery test: Six total phenolic acid samples of LJP were weighed and added to the standard solution with known concentration for detection, and the average recovery rate and relative standard deviation (RSD) value of each component were calculated. The results showed that the average spike recovery values of neochlorogenic acid, chlorogenic acid, cryptochlorogenic acid, isochlorogenic acid B, isochlorogenic acid A and isochlorogenic acid C were 99.03%, 101.1%, 99.07%, 101.43%, 98.97% and 99.86% respectively. The RSD values were 2.36%, 2.01%, 1.96%, 2.41%, 2.30% and 2.56% respectively, and the data are shown in Table S2.

1.5. Determination content of LJP

The contents of three randomly selected LJP samples were quantitatively analyzed according to the above method. The peak area value was substituted into the regression equation, and the average content of LJP was 832.88 mg/g. The relative content of the six components was neochlorogenic acid (1.92%), chlorogenic acid (59.65%), cryptochlorogenic acid (5.37%), isochlorogenic acid B (3.80%), isochlorogenic acid A (19.66%), isochlorogenic acid C (9.60%), with a purity of 83.29%.

Antibody information

The antibody information is shown in Table S3.

Figure and table legends

Figure S1. Chemical structures of the main constituents in LJP: (A) neochlorogenic acid, (B) chlorogenic acid, (C) cryptochlorogenic acid, (D) isochlorogenic acid B, (E) isochlorogenic acid A, and (F) isochlorogenic acid C.

Figure S2. HPLC chromatograms of (A) standard samples and (B) LJP. (1. neochlorogenic acid, 2. chlorogenic acid, 3. cryptochlorogenic acid, 4. isochlorogenic acid B, 5. isochlorogenic acid A, 6. isochlorogenic acid C).

Table S1 Regression equations and linear ranges of LJP.

Table S2 Spike recoveries of LJP.

Table S3 Antibody information.

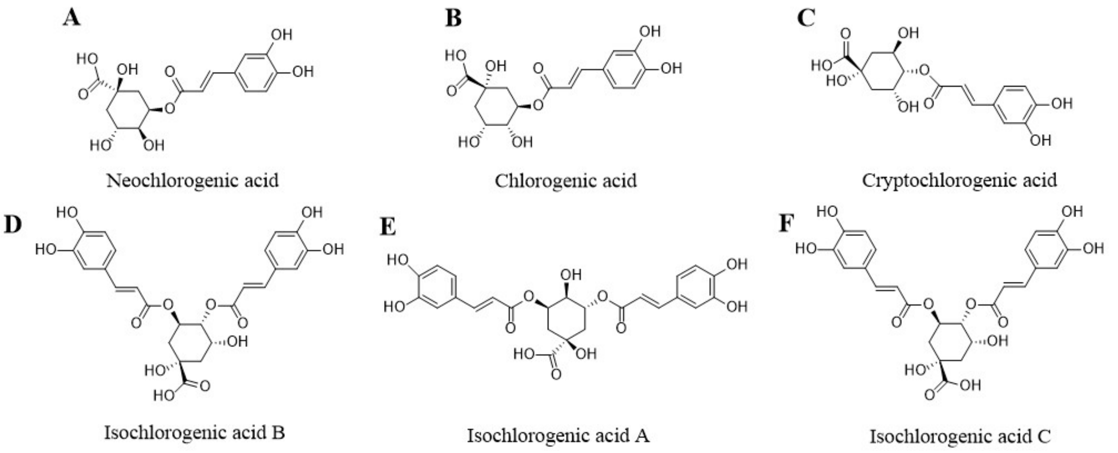

Figure S1. Chemical structures of the main constituents in LJP: (A) neochlorogenic acid, (B) chlorogenic acid, (C) cryptochlorogenic acid, (D) isochlorogenic acid B, (E) isochlorogenic acid A, and (F) isochlorogenic acid C.

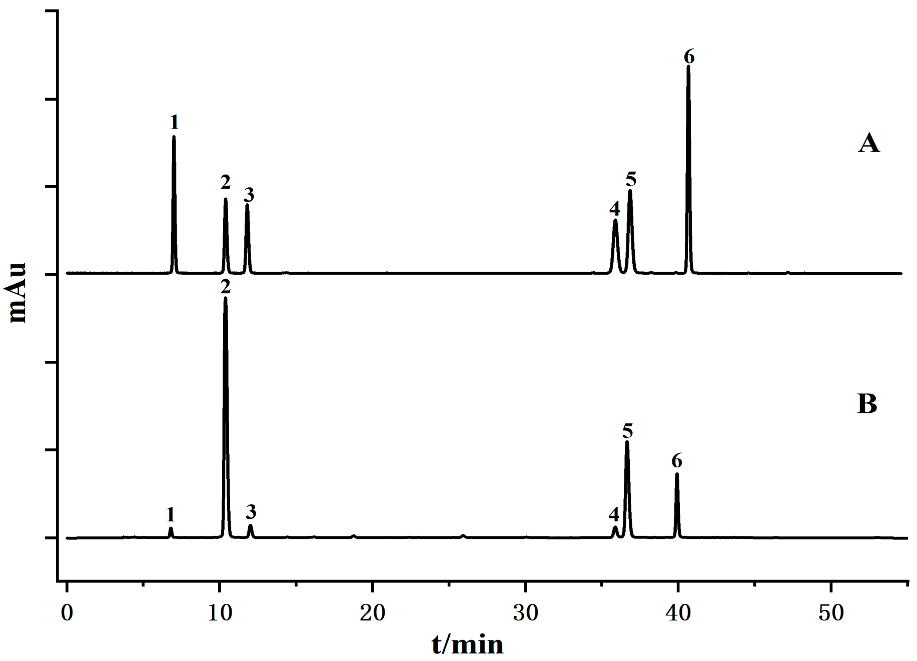

Figure S2. HPLC chromatograms of (A) standard samples and (B) LJP. (1. neochlorogenic acid, 2. chlorogenic acid, 3. cryptochlorogenic acid, 4. isochlorogenic acid B, 5. isochlorogenic acid A, 6. isochlorogenic acid C).

Table S1. Regression equations and linear ranges of LJP.

Components	Regression equation	R^2	Linear range (mg/mL)
Neochlorogenic acid	y = 7156.0 x - 202.44	0.9997	0.10～2.00
Chlorogenic acid	y = 5903.3 x - 296.57	0.9997	1.00～6.00
Cryptochlorogenic acid	y = 4283.8 x - 103.34	0.9997	0.25～2.00
Isochlorogenic acid B	y = 7147.7 x - 427.19	0.9994	0.25～2.00
Isochlorogenic acid A	y = 9100.4 x - 329.67	0.9996	0.25～2.00
Isochlorogenic acid C	y = 8398.0 x - 417.83	0.9992	0.25～2.00

Table S2. Spike recoveries of LJP.

Components	Original content /mg	Addition content /mg	Measured content /mg	Spike recovery /%	Average value /%	RSD/ %
neochlorogenic acid	0.1269	0.0638	0.1881	95.92	99.03	2.36
	0.1279	0.0638	0.1903	97.81		
	0.1274	0.0638	0.1924	101.88		
	0.1263	0.0638	0.1906	100.78		
	0.1269	0.0638	0.1890	97.34		
	0.1272	0.0638	0.1913	100.47		
chlorogenic acid	3.9413	1.8575	5.8750	104.10	101.10	2.01
	3.9721	1.8575	5.7943	98.10		
	3.9592	1.8575	5.8158	99.95		
	3.9392	1.8575	5.8385	102.25		
	3.944	1.8575	5.8217	101.09		
	3.9509	1.8575	5.8290	101.11		
Cryptochlorog -enic acid	0.3548	0.1785	0.5290	97.59	99.07	1.96
	0.3576	0.1785	0.5391	101.68		
	0.3564	0.1785	0.5338	99.38		
	0.3546	0.1785	0.5340	100.50		
	0.3551	0.1785	0.5317	98.94		
	0.3557	0.1785	0.5276	96.30		
isochlorogenic acid B	0.2511	0.1262	0.3793	101.58	101.43	2.41
	0.253	0.1262	0.3808	101.27		
	0.2522	0.1262	0.3815	102.46		
	0.2509	0.1262	0.3742	97.70		
	0.2513	0.1262	0.3780	100.40		
	0.2517	0.1262	0.3844	105.15		
isochlorogenic acid A	1.299	0.6542	1.9269	95.98	98.97	2.30
	1.3092	0.6542	1.9660	100.40		
	1.3049	0.6542	1.9616	100.38		
	1.2983	0.6542	1.9365	97.55		
	1.2999	0.6542	1.9669	101.96		
	1.3022	0.6542	1.9404	97.55		
isochlorogenic acid C	0.6343	0.3193	0.9420	96.37	99.86	2.56
	0.6393	0.3193	0.9597	100.34		
	0.6372	0.3193	0.9545	99.37		
	0.634	0.3193	0.9592	101.85		
	0.6347	0.3193	0.9647	103.35		
	0.6359	0.3193	0.9484	97.87		

Tables S3. Antibody information.

Antibodies information (lot number,company, dilution ratio)
Anti-iNOS (13120T, Cell Signaling Technology, 1:1000)
Anti-COX-2 (4842S, Cell Signaling Technology, 1:1000)
Anti-TLR4 (GB11519-100, Servicebio, WB:1:1000, IF: 1:500)
Anti-MyD88 (GB111554-100, Servicebio, WB: 1:1000, IF: 1:1500)
Anti-IKK-α (2682S, Cell Signaling Technology, 1:1000)
IKKα/β Phosphorylated antibody (2697T, Cell Signaling Technology, 1:1000)
Anti-IκBα (9242S, Cell Signaling Technology, 1:1000)
IκBα Phosphorylated antibody (2859T, Cell Signaling Technology, 1:1000)
Anti-NFκB p65 (8242T, Cell Signaling Technology, 1:1000)
NFκB p65 Phosphorylated antibody (76778SF, Cell Signaling Technology, 1:1000)
Anti-Sirt3 (10099-1-AP, Proteintech, WB: 1:5000)
Anti-GPX4 (67763-1-Ig, Proteintech, WB : 1:2000, IF: 1:500)
Anti-HO-1 (00155003, Proteintech, 1:2000)
Anti-KEAP1 (8047S, Cell Signaling Technology, 1:1000)
Anti-NRF2 (20733S, Cell Signaling Technology, WB : 1:1000, IF: 1:50)
Anti-SLC7A11 (26864-1-AP, Proteintech, 1:1000)
Anti-NQO-1 (11451-1-AP, Proteintech, 1:1500)
Anti-ZO-1 (21773-1-AP, Proteintech, 1:5000)
Anti-Claudin-1 (28674-1-AP, Proteintech, 1:2000)
Anti-Occludin (27260-1-AP, Proteintech, 1:5000)
Anti-β-actin (TA-09, ZSJB-BIO, 1:1000)
HRP-labeled goat anti-mouse IgG (GB23301, Servicebio, 1:10000)
HRP-labeled goat anti-rabbit IgG (GB23303, Servicebio, 1:10000)
